# Should obesogenic- effect be replaced with adipose tissue related alterations in childhood?

**DOI:** 10.1186/s13052-022-01210-z

**Published:** 2022-02-17

**Authors:** Angelika Anna Mohn, Nella Polidori

**Affiliations:** Department of Pediatrics, University of Chieti, Chieti, Italy

## Abstract

During the last year, primary prevention programs for childhood obesity have not obtained the goal in decreasing the prevalence of obesity in pediatric population. This phenomenon remains a crucial issue for the future translating itself in major health problems for the next young-adult generation. However ectopic adipose tissue distribution shows the same obesogenic effect also in slim people. Therefore, the use of adequate language is essential to develop consciousness of overall healthy lifestyle throughout the population. We therefore propone to replace “obesogenic effect” with adipose tissue related alterations also in childhood population.

The cornerstone of each single primary prevention program is education especially in pediatrics. A pivotal role for the success of education is played undoubtedly by an appropriated use of language which needs to be simple, self-explicative and identificatory [1]. Although largely implemented, primary prevention programs for childhood obesity have not reached their goal in reducing the prevalence of obesity in children and adolescents. This phenomenon has widely been documented not only in western countries but also in the developing world [2] remaining a crucial issue for the future and translating itself in major health problems for the next young-adult generation [3].

A substantial obstacle for obtaining good results for primary prevention programs are current environmental influences on lifestyle promoting excessive intake of pro-atherosclerotic foods combined with substantial lack of physical activity mainly due to technology over use. This leads consequently to a continuous increase of body weight through society. The recent COVID-19 restriction abolishing movement of the population almost completely together with promotion of over-the-counter food also at home through delivery systems might explicate a clear atherosclerotic and pro-diabetogenic effect [4]. These metabolic alterations seen typically in obese subjects are known under the name of obesogenic effect. However, it has been well documented that this metabolic derangement is not induced by body weight itself but more importantly by the distribution of body adipose tissue. In this respect slim people might show, to a smaller degree, the same ectopic adipose tissue distribution of obese subjects, especially intra- abdominal fat explicating the same obesogenic effect [5]. In this respect the last two COVID-19 years might consistently contribute to further metabolic derangements throughout the population including childhood. In this context together with the increasing awareness that body shaming which is particular harmful in adolescents it might be more correct to introduce the concept of adipose tissue related alterations instead of obesogenic effect. In fact, recently, the position statement of EASO proposed the new concept of “adiposity based chronic disease” which reflects both the underlying pathophysiology and the clinical impact of obesity as an established chronic disease in adults [6, 7]. This concept should also be strongly promoted for the obese children. In contrast the diagnosed term "adipose tissue related alterations" seem to be more appropriated for the overall childhood population as it helps to shift the focus from the sole obese subject to the whole population leading to different positive consequences. The first would be a prompt diagnosis and therapy of adipose tissue related metabolic alterations also in slim people. But most importantly the use of an explicative and understandable language would facilitate public education and help to develop consciousness of overall healthy lifestyle opening a window of hope for the success of primary prevention programs for adipose tissue related alterations especially in childhood.

## Data Availability

Not applicable.
